# The Influence of the Precursor’s Nature and Drying Conditions on the Structure, Morphology, and Thermal Properties of TiO_2_ Aerogels

**DOI:** 10.3390/gels8070422

**Published:** 2022-07-06

**Authors:** Jolanta Donėlienė, Eglė Fataraitė-Urbonienė, Nina Danchova, Stoyan Gutzov, Juras Ulbikas

**Affiliations:** 1 Applied Research Institute for Prospective Technologies, Vismaliuku Str. 34, LT 10243 Vilnius, Lithuania; egle.fataraite@ktu.lt (E.F.-U.); ulbikas@met.lt (J.U.); 2 JSC Modern E-Technologies, Vismaliuku Str. 34, LT 10243 Vilnius, Lithuania; 3 Faculty of Mechanical Engineering and Design, Kaunas University of Technology, Studentu Str. 56, LT 51424 Kaunas, Lithuania; 4 Department of Physical Chemistry, Faculty of Chemistry and Pharmacy, Sofia University “St. Kliment Ohridski”, J. Bourchier Blvd. 1, 1164 Sofia, Bulgaria; fhndd@chem.uni-sofia.bg (N.D.); sgutzov@chem.uni-sofia.bg (S.G.)

**Keywords:** TiO_2_ aerogel, sol–gel synthesis, solvent exchange, ageing, subcritical drying, thermal conductivity

## Abstract

A cost-effective solution for the synthesis of high-porosity TiO_2_ aerogels, which can be used as a mesoporous perovskite network charge-carrier material during the manufacture of solar cells, is described. The effects of the synthesis parameters (precursor (titanium (IV) isopropoxide (TIP) and tetrabutyl orthotitanate (TBOT)), additional solvent exchange (n-hexane (nH), cyclohexane (CH), and diethyl ether (DE)), subcritical drying (800 mbar vacuum, 70 °C, 8 h), aging, and calcination on the aerogel’s structure have been investigated. Methods of XRD, FT-IR, BET, Raman, STA, SEM, UV–vis, and thermal conductivity measurements were applied to find out the relation between the synthesis conditions and the properties of the synthesized aerogels. Amorphous aerogels are polydispersed systems with the highest probability of pore diameter from 0.5 to 15 nm. An nH-exchanged, aged aerogel synthesized from the precursor TIP shows the highest diameter of pores. After calcination, the aerogels tend to crystallize into an anatase phase and the size of the crystallites depends on the precursor’s nature. Calcination leads to a significant increase in both the apparent and true density of the aerogels, and it also results in an increase in porosity and thermal conductivity.

## 1. Introduction

Aerogels are three-dimensional solid materials with the following characteristics: pore sizes ranging from 2 to 50 nm, a very high open porosity, and a specific surface area [1]. Aerogels have some variations, such as organic aerogels (cellulose [2], etc.), carbon aerogels [3] (usually carbonized from organic aerogels), and oxide aerogels (silica [4], alumina [5], zirconia [6], and titania [7]). Titanium dioxide (TiO_2_) is considered one of the most well-known photocatalysts due to its efficient photocatalytic activities [8], low cost, and safety for human health [9] and the environment [10]. Titania can also be used in other applications, such as photocatalytic water splitting, removal of pollutants from the environment [11,12], solar cells [13], rechargeable batteries [14], sensors, supercapacitors, and biomedical devices [13,15]. In particular, for solar cell applications, a mesoporous TiO_2_ with a large specific surface area is essential to increase the amount of dye adsorption [16,17] or perovskite adsorption, as well as the power conversion efficiency [18,19].

Titania can be synthesized using different methods, such as dry methods (flame synthesis and chemical vapor deposition), wet chemistry methods (the chloride (TiCl_4_) method and hydrothermal processing) [12], and sol–gel methods [20]. Moreover, different techniques can be used for wet gels obtained by sol–gel method drying, which include the following: supercritical drying [7,20,21], freeze drying [22], post-pyrolysis, ambient drying [23], and subcritical drying [24].

The synthesis routes of the mesoporous TiO_2_ oxide involve many process parameters that can determine the mesoporous structure (i.e., Ti-alkoxide concentration, pH, surrounding media, aging time [25], solvents, etc.) [26]. Gel aging can increase cross-linking and can result in the creation of aerogels with excellent pore sizes, as well as large surface areas [27]. For the additional solvent exchange, different solvents with low surface tensions can be used, which can lower the surface tension of the pore liquid of the hydrogel. Surface tension can be decreased by applying a solvent exchange and, therefore, preventing capillary pressure from damaging the aerogel network during the drying process [28].

Understanding the mechanism of the synthesis route and determining the optimal synthesis parameters allows for selecting the most cost-effective set of aerogel synthesis conditions at which the required structural and morphological properties are obtained. In previous work [29], it was found that subcritical dried samples (400 mbar, 70 °C, 8 h) show an amorphous structure, which after calcination tends to crystallize in the anatase phase and affect the microstructure of the aerogels. The originally obtained aerogels show structural and optical properties comparable to TiO_2_ structures mentioned in the literature and appear to be quite promising for photovoltaic applications.

This investigation focuses on cost-effective solutions for the synthesis of high-porosity TiO_2_ aerogels, which can be used as mesoporous perovskite network charge-carrier materials for solar cell production. The next step and goal of the investigation was to evaluate the influence of lowering down the vacuum to 800 mbar on the properties of TiO_2_ aerogels. The influence of the precursor type, gel aging, additional solvent exchange, and other synthesis parameters that control the formation of the TiO_2_ aerogels has also been investigated.

## 2. Results and Discussion

To understand the effects of the synthesis conditions on the structural characteristics of TiO_2_ aerogels, different analysis methods, such as X-ray diffraction (XRD), Fourier-transform infrared spectroscopy (FT-IR), Brunauer–Emmett–Teller (BET), Raman spectroscopy, simultaneous thermal analysis (STA), scanning electron microscopy (SEM), ultraviolet–visible (UV–vis) spectroscopy, and thermal conductivity measurements, were applied to identify and discuss trends for further aerogel structural optimizations.

### 2.1. XRD Analysis

Figure S1 shows XRD patterns that characterize the structure of synthesized subcritical dried (800 mbar, 70 °C, 8 h) TiO_2_ aerogels in relation to the nature of the precursor (titanium (IV) isopropoxide (TIP) and tetrabutyl orthotitanate (TBOT)), the type of solvent used for the additional exchange (n-hexane (nH), cyclohexane (CH), diethyl ether (DE)), and the effects of ageing (72 h, 40 °C). All investigated TiO_2_ aerogel samples show an amorphous structure [30] (Figure S1).

### 2.2. FT-IR Analysis

**Figure 1:**
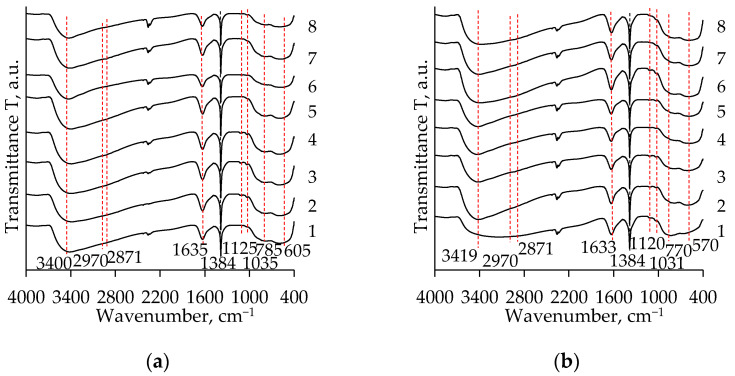
FT-IR spectra of the TiO_2_ aerogels after subcritical drying (800 mbar, 70 °C, 8 h) without aging (1–4) and after 72 h of aging (5–8) depending on the precursor type (TIP (**a**), TBOT (**b**)) and the solvent used for an additional solvent exchange (1, 5—without exchange; 2, 6—CH; 3, 7—nH; 4, 8—DE).

The FT-IR spectra of the TiO_2_ aerogels are presented in Figure 1. When comparing the obtained curves, it can be stated that significant differences between them do not occur. The characteristic peaks between 400 and 1000 cm^−1^ can be attributed to the bending vibration of the Ti–O–Ti and the stretching vibration of the Ti–O bonds [1,8,31]. These broad peaks in the mentioned range could also be attributed to a combination of Ti–O–Ti and Ti–O–C bond stretching vibrations [11].

The weak absorption peaks at 1040–1120 cm^−1^ can be attributed to the stretching vibration of the C–C bonds [21,31]. The peak at 1380 cm^−1^ is due to the symmetric deformation vibration of CH_3_ [21]. The peaks at 2970 and 2871 cm^−1^ can be assigned, respectively, to symmetric CH_2_ and CH_3_ stretching [31]. Typically, these peaks characterize the remaining organic compounds (in this case, they can be dissociative organic solvents (nH, CH, and DE) and EtOH) in the pores of the TiO_2_ aerogels [8,31]. The wide absorption bands at ~1633 cm^−1^ and ~3390 cm^−1^ can be related to the bending vibrations of the adsorbed water molecules (H–O–H) and the stretching vibrations of the hydroxyl (OH) groups on the surface of the aerogel, respectively [1,8,31].

### 2.3. BET Analysis

**Figure 2:**
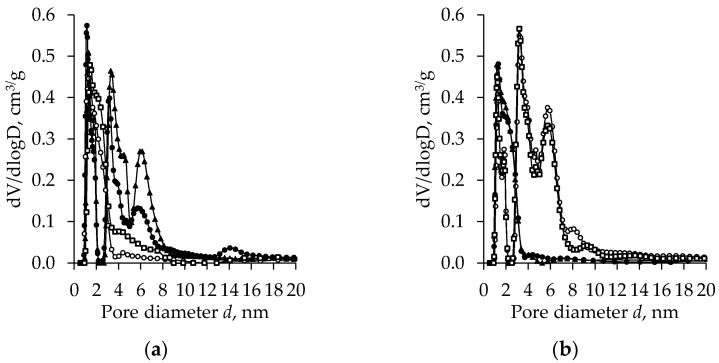
The pore size distribution of subcritical dried (800 mbar, 70 °C, 8 h) TiO_2_ aerogels synthesized using the TBOT precursor versus the type of exchange solvent (●—without exchange, ○—nH, ▲—CH, □—DE) and ageing (without (**a**) and after (**b**) ageing).

**Figure 3:**
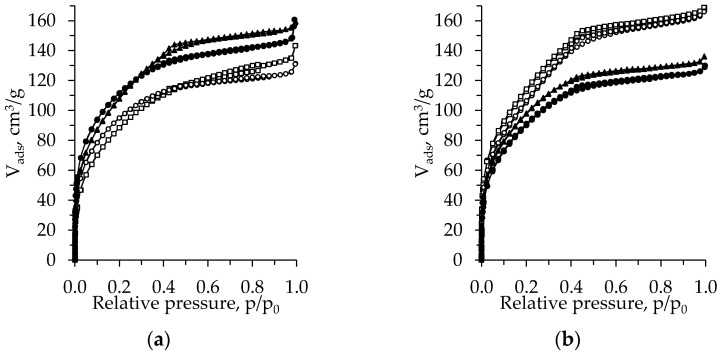
Isotherms of the subcritical dried (800 mbar, 70 °C, 8 h) TiO_2_ aerogels (precursor TBOT) versus the type of exchange solvent (●—without exchange, ○—nH, ▲—CH, □—DE) and ageing (without (**a**) and after (**b**) ageing).

**Figure 4:**
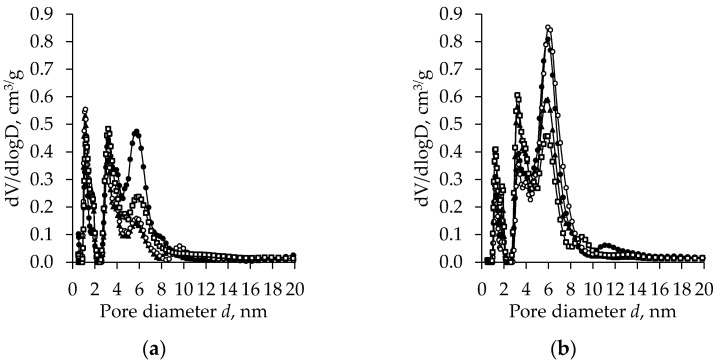
The pore size distribution of the subcritical dried (800 mbar, 70 °C, 8 h) TiO_2_ aerogels (precursor TIP) vs. the type of exchange solvent (●—without exchange, ○—nH, ▲—cH, □—DE) and ageing (without (**a**) and after (**b**) ageing).

**Figure 5:**
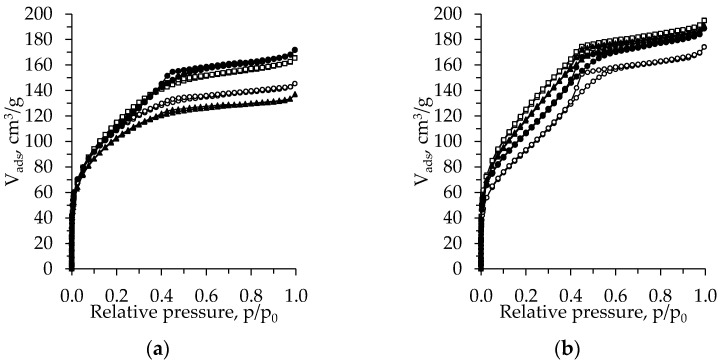
Isotherms of the subcritical dried (800 mbar, 70 °C, 8 h) TiO_2_ aerogels (precursor TIP) vs. the type of exchange solvent (●—without exchange, ○—nH, ▲—CH, □—DE) and ageing (without (**a**) and after (**b**) ageing).

Figure 2, Figure 3, Figure 4 and Figure 5 show the results of BET analysis of unaged and aged subcritical dried (800 mbar, 70 °C, 8 h) TiO_2_ aerogels synthesized using the precursor TBOT or TIP together with an additional solvent exchange in CH, nH, or DE. In all cases, the aerogels are polydisperse systems, the characteristics of which depend on the synthesis conditions, the precursor nature, and the type of solvent used for the additional exchange.

Figure 2 shows pore size distribution curves of the unaged and aged TiO_2_ aerogels obtained using the precursor TBOT and additionally exchanged in different solvents. For the unaged and without an additional exchange-synthesized aerogels, the pore diameter distribution curve has four peaks. The highest peak was found at 1 and 4.0 nm, twice as low at 6 nm, and the lowest peak was found at 14 nm. The character of the pore size distribution curve changes substantially after the additional solvent exchange. In the case when CH is used, the first three peaks remain in the pore distribution curves with their peaks being in the same position as the samples without an additional exchange, but their height is lower. The heights of the first and second peak are about twice as low and those of the third peak are about 1.4 times lower compared to the samples for which an additional exchange was not applied. The additional solvent exchange with DE and nH results in radical changes in the pore distribution mode. The unimodal pore size distribution indicates a decrease in the polydispersity of the system. The highest probability of the diameter of the pores in these aerogels is 0.5–3 nm, and the number of the larger pores in these aerogels is insignificant.

Aging changes the nature of the pore size distribution curves for these aerogels (Figure 2b). The pore distribution curves have been changed from polymodal to unimodal with a peak in the range from 1 to 4 nm for the aerogels without an additional exchange or additionally CH-exchanged. Compared to the unaged samples without an additional exchange, the peak height after aging was 2-fold decreased, but the width of the peak increased. After the aerogels were aged and additionally exchanged in the solvents nH and DE, a wider pore size distribution was observed and the mode of the curves was changed from unimodal to curves with three clear expressed peaks. In this case, the effect of the solvent is not efficient as the mode of the curves is very similar. Here, an increase in the number of pores with diameters ranging from 2 to 8 nm is observed.

Figure 3 shows the N_2_ adsorption–desorption isotherms of the TiO_2_ aerogels from the precursor TBOT, dried under subcritical conditions (800 mbar, 70 °C, 8 h).

The isotherms of the unaged aerogels can be classified as type I with an H4 hysteresis loop. The exception is the CH-exchanged sample, the isotherm of which can be attributed to the type IV isotherm with an H2 hysteresis loop (Figure 3a) [32,33]. After aging, the isotherm of the CH-exchanged sample changes to type I isotherms with an H4 hysteresis loop and that of the nH- and DE-exchanged aerogels to type IV isotherms with an H2 hysteresis loop. For the samples without an additional exchange isotherm type, the hysteresis loop remains the same, while the adsorption values decrease (Figure 3b). The data coincide well with those of the pore size distribution curves (Figure 2).

Three characteristic peaks in the zone from 0–8 nm were observed in the pore size distribution curves of the unaged subcritically dried TiO_2_ aerogels synthesized from the precursor TIP (Figure 4a). For the first two peaks, there is no significant influence of the solvent type used for the additional exchange. The peak height decreases slightly with the increasing pore diameter. A more pronounced effect of the additional solvent exchange was observed for the third peak. After the additional nH exchange, the height of the third peak at 6 nm decreased more than twice and a small fourth peak at 9 nm occurred.

The curves of the aged and unaged samples show that aging changes the porosity of the TiO_2_ aerogels (Figure 4b). After aging, the nature of the distribution curves of the TiO_2_ aerogels synthesized from the precursor TIP remains the same (the curves have three peaks), but there are significant differences in their height. The height of the first peak (nanopore diameter was about 1.0 nm) decreases from 0.6 down to 0.4, the height of the second peak remains virtually unchanged, and the third peak height increases to 0.8 at 6.0 nm. The influence of the solvent used for the additional exchange is also evident. For additionally nH-exchanged samples, the amount of pores with a diameter of 6 nm is higher than for the samples without an additional exchange, for which additional exchanges in other solvents have been used. In other cases, the size and amount of the pores were close to those of the unaged specimens.

Figure 5 shows the N_2_ adsorption–desorption isotherms of the TiO_2_ aerogels obtained using the precursor TIP. The isotherm of the unaged TiO_2_ aerogel without an additional solvent change can be classified as type IV isotherms with an H2 hysteresis loop and the remaining samples can be classified as type I isotherms with an H4 hysteresis loop (Figure 5a). After aging, the isotherms of all obtained aerogels are changed to type IV with an H2 hysteresis loop (Figure 5b) [32,33].

The influence of aging on the TiO_2_ aerogel pore size distribution has been found to be highly dependent on the precursor’s nature; however, it is difficult to identify a clear effect of the solvent type used for the additional exchange. It is interesting to state that the same tendency was found under subcritical drying conditions when a 400 mbar pressure was applied [29]. A summary of the BET analysis results is provided in Table 1.

### 2.4. Raman Analysis

**Figure 6:**
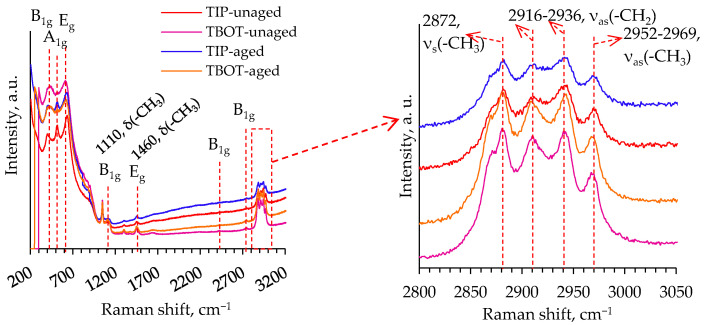
Raman spectra of the unaged and aged TiO_2_ aerogels synthesized without an additional solvent exchange.

Figure 6 shows the Raman spectra of the original prepared unaged and aged TiO_2_ aerogels without an additional solvent exchange. The observed peaks in the Raman shift of 400, 515, and 640 cm^–1^ can be ascribed to the characteristic vibrational modes B_1g_(1), A_1g_(2), and E_g_(3) for the anatase phase of the TiO_2_. Independently of the precursor’s nature, the Raman peaks also appear at the Raman shift, exceeding 800 cm^−1^. The peaks at 1110–1460, 2872, 2916–2936, and 2952–2969 cm^–1^ can be attributed to the –CH_3_ bending vibration mode, –CH_3_ stretching mode, –CH_2_ asymmetric stretching mode, and the –CH_3_ asymmetric stretching mode, respectively [34,35].

The results obtained are in a good agreement with the FT-IR and STA results. It is confirmed that after subcritical drying, residues of organic compounds remain in the structure or the pores of the TiO_2_ aerogels. The Raman spectra analysis used here shows the formation of the anatase phase in the TiO_2_ aerogels.

### 2.5. Thermal Analysis

**Figure 7:**
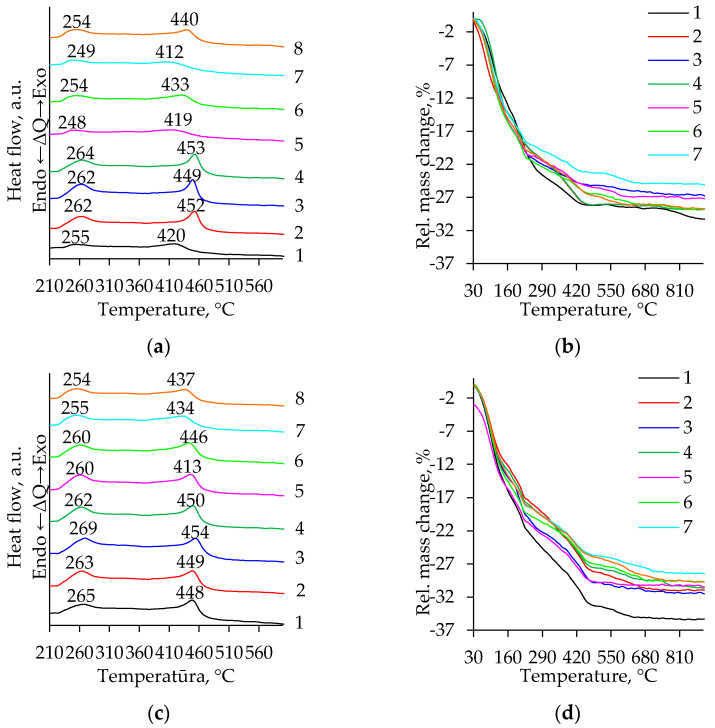
DTA (**a**,**c**) and TG (**b**,**d**) curves of the TiO_2_ aerogels after subcritical drying (800 mbar, 70 °C, 8 h) vs. precursor type (TIP (**a**,**b**), TBOT (**c**,**d**)), ageing (without aging (1–4), aged (5–8)), and additional solvent exchanges (1, 5—without exchange; 2, 6—CH; 3, 7—nH; 4, 8—DE).

In Figure 7, the typical results of the simultaneous thermal analysis (differential thermal (DTA)—Figure 7a,c; thermogravimetric (TG) analysis—Figure 7b,d) of the synthesized aerogels are presented. It is evident that for all DTA curves in the temperature region from 200 °C to 500 °C two peaks are characteristic. The results of these curves are summarized in Table S1 and Table 2. The first exothermic peak in Figure 7a,b (Table S1) starts at ∼204 °C and continues to ~309 °C and the obtained weight loss (∼2.2–6.6%) in this temperature range is probably due to the reaction and the release of residual organic compounds (excluding solvents and alkoxy groups) [36,37,38]. The second exothermic peak at 368–474 °C can be attributed to the formation of the anatase (Figure 7c,d; Table 2), i.e., the conversion of Ti(OH)_4_ into TiO_2_ (dehydroxylation) and the occurred crystallization of the anatase [38,39]. The materials do not suffer any thermal changes above 500 °C. A mass loss below ~225 °C in the TG curves can be attributed to adsorbed impurities and moisture [36,39].

The total mass loss was between 25–33% and 27–36% for the samples prepared using the precursors TIP (Figure 7a, Table S1) and TBOT (Figure 7b, Table S1), respectively. For comparison, after the high-temperature supercritical drying, the mass loss in the TG curves is approximately only up to ~5% [40] and this tends to suggest that for supercritical dried samples the decomposition of the organic groups is already reached.

**Figure 8:**
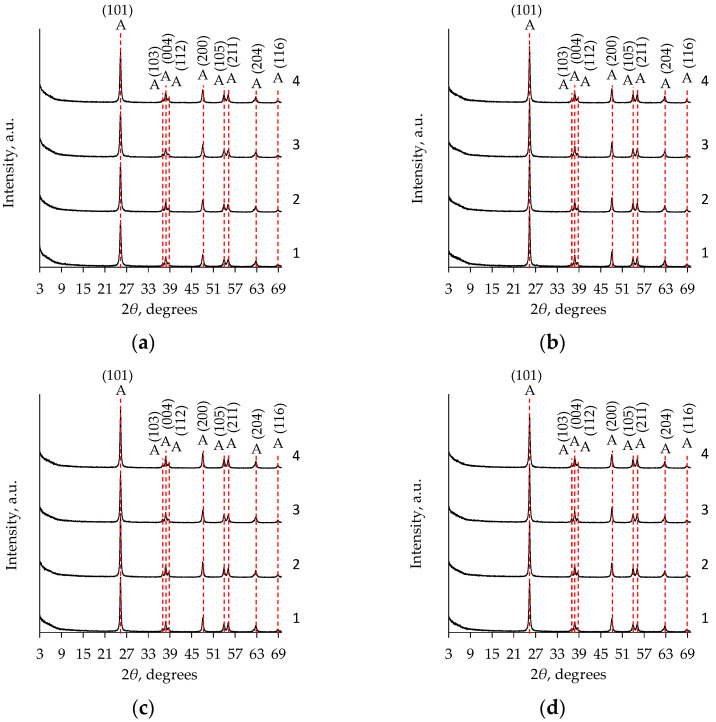
The effects of calcination on the TiO_2_ aerogel XRD patterns after subcritical drying (800 mbar, 70 °C, 8 h) in relation to the precursor (TIP (**a**,**b**), TBOT (**c**,**d**)), ageing (without aging (**a**,**c**), aged (**b**,**d**)), and additional solvent exchanges (1—without exchange; 2—CH; 3—nH; 4—DE). Indices: A—anatase phase.

To determine whether the exothermic peak at 368–474 °C in the DTA curves was induced by the formation of the anatase phase, several samples were heat-treated at 500 °C for two hours. This temperature was chosen based on data from the literature [41] and the obtained results (Figure 7, Table 2) are as follows: the anatase phase is formed at a temperature higher than 400 °C. Moreover, the selected calcination temperature must ensure full thermal conversions in the mentioned temperature range. To confirm this, the calcinated samples were investigated by XRD analyses (Figure 8). All calcinated TiO_2_ aerogel samples crystallized in the anatase phase (PDF-00-064-0863) [30]. In all investigated cases, the relative intensity of the peaks in the XRD patterns (Figure 8) shows a similar intensity.

For comparison, Table 3 presents the size of the crystallites for aged and unaged TiO_2_ aerogels synthesized without a solvent exchange and additionally nH-exchanged. No significant crystallite size was found when the synthesis was performed using the precursor TBOT, while aging resulted in an increase in the crystallite size for the TiO_2_ aerogels synthesized from TIP.

### 2.6. Morphology of TiO_2_ Aerogels

**Figure 9:**
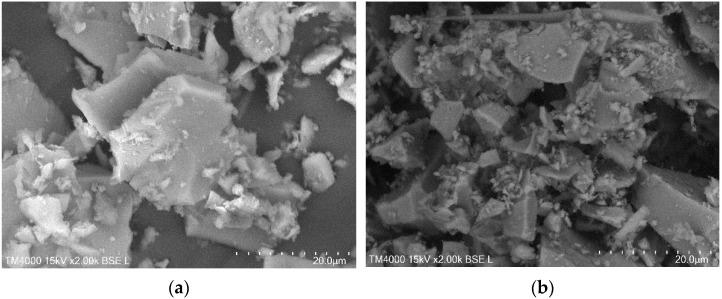
SEM micrographs of aged TiO_2_ aerogel (precursor TIP) powders: (**a**) before calcination; (**b**) after calcination.

The SEM investigation results show a typical morphology for micrometer-designed aerogel-like powders (Figure 9). Micrometer-sized particles are visible in the samples [12,31,42]. The particles are highly polydispersed and the smallest ones of these are agglomerated. It seems that the particle size tends to decrease after calcination, most probably because of the anatase phase formation.

Calcination also results in an increase in both the apparent (*ρ*_a_) and true (*ρ*_t_) density of the aerogels and in a small increase in porosity (Table 4).

### 2.7. UV–Vis Spectroscopy Analysis

**Figure 10:**
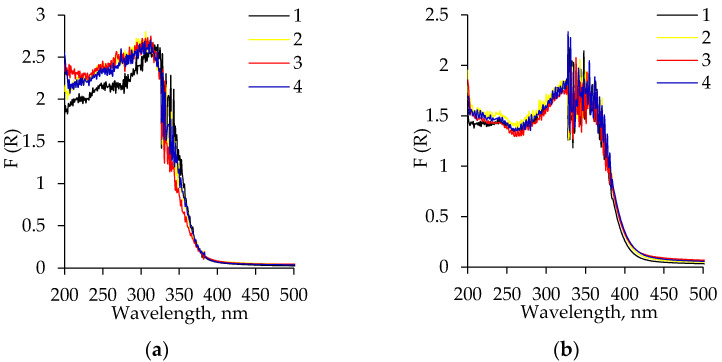
Diffuse reflectance spectra of the aged TiO_2_ aerogels: (**a**) without calcination; (**b**) after calcination. 1–2 correspond to the precursor TIP and 3–4—to TBOT: 1, 3—without solvent exchange; 2, 4—nH-exchanged.

The optical properties of the synthesized aerogels were evaluated by the UV–vis spectroscopy method. All obtained spectra show a strong UV absorption with a shoulder at about 335–340 nm, which is typical for catalytic titania derivatives (Figure 10a).

**Figure 11:**
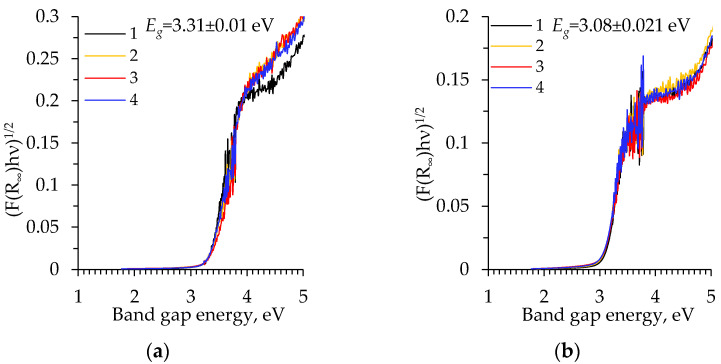
Tauc plot of the aged TiO_2_ aerogels: (**a**) without calcination; (**b**) after calcination. 1–2 curves correspond to the precursor TIP and 3–4—to TBOT: 1, 3—without solvent exchange; 2, 4—nH-exchanged.

The calcination leads to a shift in the optical bandgap with 30 nm or longer wavelengths (Figure 10b) [43,44], which are responsible for an increase in the thermal conductivity (see next section). Surface defects in titania realized by surface diffusion or cationic and anionic doping often lead to a weak coloration or a bandgap shift of ceramic oxide powders [45,46]. The optical bandgap energy *E_g_* of the original and calcinated samples was calculated using the Tauc plot method (Figure 11) [47].

The investigated amorphous TiO_2_ aerogels showed a higher indirect bandgap energy (*E_g_* = 3.31 ± 0.01 eV, Figure 11a) compared to the crystalline phase *E*_g_, which is equal to 3.08 ± 0.028 eV (Figure 11b) [48]. The following results are in a good agreement with the data from the literature: the anatase, as an indirect bandgap semiconductor, has a bandgap energy between 3 and 3.2 eV [8,10,44]. These values are very close to those found for the subcritically dried aerogels at a 400 mbar vacuum. [29]. The indirect bandgap energy (*E_g_*) of the uncalcinated samples dried at a 400 mbar vacuum was determined to be equal to 3.32 ± 0.037 eV and for the calcinated samples it was 3.08 ± 0.026 eV. Moreover, the anatase bandgap can be changed by variations in the size of the nanoparticles or the synthesis conditions [11].

### 2.8. Thermal Conductivity Measurements

The results of the thermal conductivity measurements are presented in Table 5. In the last column of Table 5, the increase in the thermal conductivity of the samples, Δ*k*, as a result of the calcination, is given. An increase in *k* is detected as a result of the calcination, depending on the initial aerogel chemistry.

The increase in Δ*k* correlates with the UV–vis measurements, where there is a decrease in the optical bandgap as a result of heating, which is equivalent to an increase in the electrical conductivity. In semiconductors and insulators, the decrease in the optical bandgap is proportional to the electrical conductivity.

The thermal conductivity (*k*) and electrical conductivity (σ) of the materials are connected by the Wiedemann–Franz law as follows:

*k*/*σ* = *LT*, (1)

where *T* is the temperature and *L* = 2.44 × 10^−8^ V^2^K^−2^ [49]. Thus, the increase in the thermal conductivity (Table 3) can be connected with the increase in the electronic contribution to the thermal conductivity.

## 3. Conclusions

A cost-effective solution for synthesizing high-porosity TiO_2_ aerogels, which can be used as mesoporous perovskite network charge-carrier materials during solar cell manufacturing, is developed. The influence of a lower vacuum (800 mbar) and the effect of titanium precursors (TIP and TBOT), solvents (nH, CH, DE) used for an additional solvent exchange, aging, and calcination on the properties of the final products have been investigated. The subcritical dried samples at an 800 mbar vacuum show an amorphous structure, which, after calcination, tends to crystallize in the anatase phase. The size of the crystallites depends on the nature of the precursor. Selected synthesis conditions result in the formation of highly polydispersed aerogels with the highest probability of pore size distribution in the region from 0.5 to 15 nm. The highest diameter of the pores has been found for the additionally nH-exchanged, aged aerogels synthesized from the precursor TIP. Calcination also results in an increase in both the apparent and true density of the aerogels and in an increase in their porosity. Independently of the precursor type, the apparent and true density of the calcinated aerogels increases twice compared to the uncalcinated species. The calcination of the aerogels results in an increase in the thermal conductivity because of an optical bandgap decrease, detected by UV–vis reflectance spectroscopy. The obtained results show that it is meaningful to evaluate the applicability of the synthesized aerogels in perovskite solar cells.

## 4. Materials and Methods

Titanium (IV) isopropoxide (TIP, 98% Fluorochem, Glossop, UK) and tetrabutyl orthotitanate (TBOT, 95% Fluorochem, Glossop, UK) were used as the titanium sources. Distilled water (H_2_O) and ethanol (EtOH, 99.5%, Emparta ASC, Merck-KGaA, Darmstadt, Germany) were used as the solvents. Nitric acid (HNO_3_, 65%, Chempur, Piekary Slaskie, Poland) was used as a catalyst, as well as a chelating agent. Ethanol, n-hexane (nH, 99%, Chempur, Piekary Slaskie, Poland), cyclohexane (CH, 99%, Chempur, Piekary Slaskie, Poland), and diethyl ether (DE, 99.5%, Chempur, Piekary Slaskie, Poland) were used for the solvent exchanges. All the chemical reagents were used as received.

### 4.1. Synthesis of TiO_2_ Aerogels

A TiO_2_ aerogel was synthesized through a sol–gel method, earlier reported by our group [29]. Drying of the gel network was performed at a subcritical condition at an 800 mbar vacuum in a vacuum oven at a temperature of 70 °C. The drying duration was 8 h. The main characteristics of the used vacuum system were as follows: chamber (VC50, SalvisLAB, Reussbühl/Lucerne, Switzerland) volume was 50 L and the vacuum system, Vacuubrand PC 8/RC 6 (Vacuubrand GMBH + CO KG, Wertheim, Germany), had a maximum pumping speed of 5.9/6.9 m^3^/h. The synthesized samples were sieved through a sieve (with a mesh width of 80 µm). The thermal treatment for part of the samples was carried out at a temperature of 500 °C for a duration of 2 h with a heating rate of 4 °C/min (SNOL 10/1300, SnolTherm business unit, part of Umega Group, AB, Lithuania).

### 4.2. Characterization

X-ray diffraction (XRD), Fourier-transform infrared spectroscopy (FT-IR), Brunauer–Emmett–Teller (BET), Raman spectroscopy, simultaneous thermal analysis (STA), scanning electron microscopy (SEM), ultraviolet–visible (UV–vis), and thermal conductivity measurements were applied to investigate and compare the properties of the synthesized TiO_2_ aerogels and to find out the most effective synthesis route.

Powder X-ray diffraction (XRD) spectra were carried out using a D8 Advance diffractometer (Bruker AXS, Karlsruhe, Germany) (CuK_α_ radiation was generated at 40 mA and 40 kV, range 2*θ* = 3–70°, a scanning speed of 6°/min, scan type—a coupled two theta/theta).

For the Fourier-transform infrared (FT-IR) spectroscopy, a Perkin-Elmer FT-IR system spectrometer (Perkin Elmer, Boston, MA, USA) using KBr tablet-shaped samples over the wave–number range from 400 to 4000 cm^−1^ (±0.01 cm^−1^) was used.

Brunauer–Emmett–Teller (BET) analysis was performed with the surface area analyzer Autosorb iQ (Quantachrome Instruments, Boynton Beach, FL, USA) using an N_2_ gas adsorption isotherm at 77 K.

The bond structure was analyzed by μ-Raman spectroscopy (Renishaw inVia spectrometer, Wotton under Edge, UK) using a 1.5 mW excitation at a wavelength of 532 nm, focused on a 4 μm spot; the exposure duration was 10 s and the accumulation quantity was 5 for the spectral range from 200–3200 cm^−1^.

An STA (differential scanning calorimetry—DSC and thermogravimetry—TG) using the Linseis instrument STA PT1000 was performed. The heating rate was 15 °C/min and the temperature range was from 30 °C to 950 °C. The test was carried out under air atmosphere using ceramic sample handlers and platinum crucibles.

For SEM, the standard electron microscope Hitachi TM 4000 working on an SE regime was used. The particles were Au-covered.

The apparent density (*ρ*_a_) was calculated by measuring the volume and mass of the aerogels. The porosity was calculated by using the apparent density and true density (*ρ*_t_) as follows:

(2) $$Porosity=\frac{\left(\rho_{t}-\rho_{a}\right)}{\rho_{t}}\cdot 100\%$$

The room temperature diffuse reflectance spectra were measured on a Perkin-Elmer (Walham, MA, USA) Lambda 35 spectrophotometer equipped with a reflectance accessory (RSA-PE-20, Labsphere, NorthSutton, NH, USA) and a vertical sample holder with a quartz glass window between 250 nm and 900 nm. As a reference, white and black certified reflectance standards Labsphere^®^ were used. The f–f transitions and UV charge transfer transitions (CTT) of Ho_2_O_3_ and Sm_2_O_3_ micropowders were used as a reference. The peak maxima and intensities in the region between 250–750 nm were in a good agreement with the theory [50,51]. From the measured diffuse reflectance R (%), the Kubelka–Munk function F(R) was calculated [52].

A Tauc plot method was used to determine the optical energy bandgap (*E_g_*) of the selected samples. The optical absorption strength depends on the difference between the photon energy and the bandgap as follows:

(*F*(*R*)*hν*)*^1^*^/*n*^ = *A*(*hν* − *E_g_*) (3)

where *h* is the Planck’s constant, *ν* is the photon’s frequency, *n*  =  2 for the indirect allowed transitions, *E_g_* is the bandgap, and *A* is the slope of the Tauc plot in the linear region [47].

The thermal measurements were performed on a C-THERM TCi thermal conductivity analyzer configured with a Modified Plane Source (MTPS) sensor using a TCI small volume test kit (SVTK) for the measurements of powders, foams, and gels.

## Figures and Tables

**Table 1:** The morphology of the TiO_2_ aerogels versus precursor, solvent type, and ageing duration.

Precursor	Ageing Duration, h	Solvent	Surface Area *, m^2^/g	Pore Volume *, cm^3^/g	C_BET_ Constant *
TIP	0	- CH nH DE	397.257 373.878 398.166 420.178	0.244 0.192 0.206 0.235	90.156 84.773 82.372 67.304
	72	- CH nH DE	396.665 439.581 351.235 462.211	0.269 0.271 0.247 0.276	55.687 47.050 45.727 55.375
TBOT	0	- CH nH DE	409.955 404.379 348.767 334.882	0.212 0.225 0.182 0.196	78.273 49.223 68.408 42.937
	72	- CH nH DE	337.288 363.361 399.636 421.080	0.183 0.192 0.237 0.240	50.619 57.696 43.530 53.418

* The difference in the measured values for the same group does not exceed 5%.

**Table 2:** Details of STA curves that are typical for TiO_2_ aerogel anatase phase formation.

Precursor	Ageing Duration, h	Solvent	Peak Characteristics			Heat of Process, J/g	Mass Loss, %
			T_onset_, °C	T_max_, °C	T_end_, °C		
TIP	0	-	380.8	420.8	449.7	283.83	2.272
		CH	428.9	452.1	467.4	678.39	3.752
		nH	429.2	449.4	462.7	706.00	4.279
		DE	430.5	452.5	468.5	705.21	3.551
	72	-	369.4	419.4	456.1	282.60	2.407
		CH	388.8	432.5	458.3	385.80	2.251
		nH	368.9	412.4	455.2	282.95	1.863
		DE	405.7	440.2	462.1	447.65	3.077
TBOT	0	-	418.4	448.8	465.9	651.85	5.063
		CH	413.1	449.4	468.0	729.47	5.118
		nH	421.2	454.3	473.2	765.44	5.140
		DE	418.7	450.1	466.8	714.02	4.809
	72	-	410.1	446.1	464.8	703.29	4.494
		CH	404.3	443.8	465.1	651.50	4.175
		nH	383.9	434.3	460.5	518.37	3.863
		DE	392.9	437.0	460.1	446.77	3.496

**Table 3:** Variation in crystallite size depending on the TiO_2_ aerogel synthesis conditions.

Precursor	Ageing Duration, h	Solvent	Crystallite Size, nm
TIP	0	-	18.52
		nH	18.03
	72	-	20.62
		nH	21.60
TBOT	0	-	22.92
		nH	20.11
	72	-	20.47
		nH	23.08

**Table 4:** TiO_2_ aerogel density and porosity in relation to the synthesis conditions.

Precursor	Ageing Duration, h	Solvent	*ρ*_a_, cm^3^/g		*ρ*_t_, cm^3^/g		Porosity, %	
			Before Calcination	After Calcination	Before Calcination	After Calcination	Before Calcination	After Calcination
TIP	72	-	0.68	1.13	2.66	4.89	74	77
		nH	0.68	1.20	-	-	-	-
TBOT	72	-	0.69	1.16	2.29	4.36	70	73
		nH	0.69	1.29	-	-	-	-

**Table 5:** Results of thermal conductivity measurements of the aged and calcinated TiO_2_ aerogel samples.

Precursor	Solvent		Thermal Conductivity *k* *, W/m·K	Δ*k* After Calcination
TIP	-	Before calcination	0.085	-
	nH		0.079	-
TBOT	-		0.084	-
	nH		0.082	-
TIP	-	After calcination	0.100	0.016
	nH		0.099	0.02
TBOT	-		0.106	0.024
	nH		0.105	0.023

* The relative standard deviation of the measurements (RSD) is 0.7%.

## Data Availability

Not applicable.

## Supplementary Materials

The following supporting information can be downloaded at: https://www.mdpi.com/article/10.3390/gels8070422/s1, Figure S1: XRD patterns of TiO_2_ aerogels after subcritical drying (800 mbar, 70 °C, 8 h) without aging (1–4) and after 72 h of aging (5–8) depending on the precursor type (TIP (a), TBOT (b)) and the solvent used for the additional solvent exchange (1, 5—without exchange; 2, 6—CH; 3, 7—nH; 4, 8—DE); Table S1: Details of STA curves that are typical for TiO_2_ aerogel organic groups decomposition.
