# Supplementary material for: The Influence of the Precursor’s Nature and Drying Conditions on the Structure, Morphology, and Thermal Properties of TiO2 Aerogels
Source: Gels. 2022 Jul 6;8(7):422. doi: 10.3390/gels8070422 (PMC9323803; doi:10.3390/gels8070422)
Supplement: Supplementary file 1 [file gels-08-00422-s001.zip › gels-1786307-supplementary.pdf]

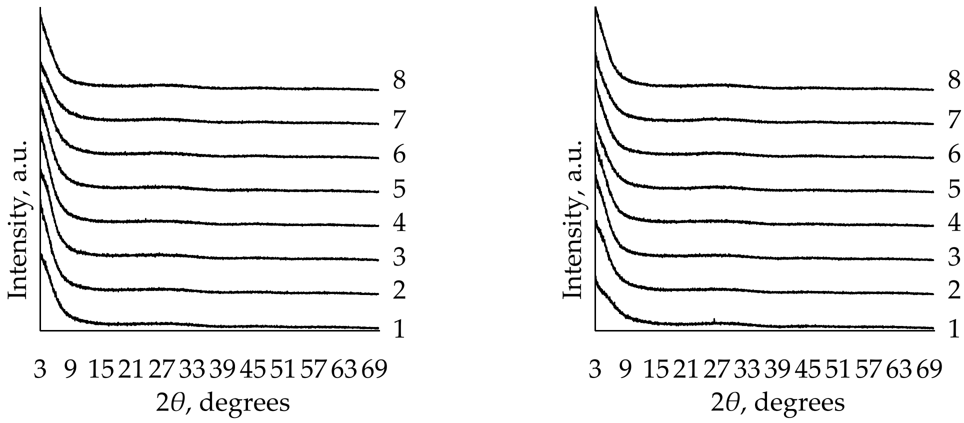

(**a**) (**b**)

Figure S1. XRD patterns of TiO_2 aerogels after subcritical drying (800 mbar, 70 °C, 8 h) without aging (1-4) and after 72 h of aging (5-8) depending on the precursor type (TIP (a), TBOT (b)) and the solvent used for additional solvent exchange (1, 5 – without exchange; 2, 6 – CH; 3, 7 – nH; 4, 8 – DE).

Table S1. Details of STA curves that are typical for TiO_2 aerogel organic groups decomposition.

Precursor	Aging duration, h	Solvent	Peak characteristics			Heat of process, J/g	Mass loss,%	Total mass loss, %
			T_{onset}, °C	T_{max}, °C	T_{end}, °C			
TIP	0	-	234.8	255.2	292.0	205.34	4.544	31.427
		CH	225.1	262.2	299.5	735.42	4.239	28.249
		nH	228.9	262.2	297.0	843.01	6.386	32.275
		DE	224.9	264.3	300.5	732.87	2.871	25.259
	72	-	231.9	247.9	288.8	199.09	2.867	27.135
		CH	229.5	254.4	299.5	376.81	2.251	29.083
		nH	230.6	248.5	288.4	261.28	2.934	25.207
		DE	227.3	254.2	294.0	492.27	3.998	28.680
TBOT	0	-	225.9	264.9	305.7	599.08	6.634	35.166
		CH	226.5	263.1	301.5	932.41	5.948	30.748
		nH	231.2	268.9	308.1	954.61	5.067	31.676
		DE	226.6	262.3	299.3	889.02	5.228	30.909
	72	-	224.5	260.4	298.4	886.73	4.771	27.560
		CH	225.0	259.8	300.0	750.26	3.655	29.826
		nH	225.2	254.5	291.2	597.11	3.825	28.518
		DE	225.3	254.1	294.6	602.89	4.336	30.118
